# 4362 The Utilization of Polyethylene Glycol Fusion to Improve Facial Reanimation

**DOI:** 10.1017/cts.2020.319

**Published:** 2020-07-29

**Authors:** Marissa Suchyta, Si-Gyun Roh, Diya Sabbagh, Mohammed Morsy, Huan Wang, Samir Mardini

**Affiliations:** 1 Mayo Clinic

## Abstract

OBJECTIVES/GOALS: This study’s goal is to determine whether intraoperative treatment of facial nerves with polyethylene glycol (PEG) fusion technology improves facial paralysis outcomes. Improved facial nerve regeneration in facial paralysis patients would lead to improved recovery time and effectiveness. METHODS/STUDY POPULATION: 30 rats were utilized; 15 underwent facial nerve regeneration without PEG fusion, and 15 with PEG fusion. Facial paralysis was initiated on the left by transection of the buccal and marginal mandibular branches of facial nerve. The buccal branch was repaired though microsuture technique. Neurorrhaphy sites of rats in the PEG group were exposed to calcium free saline, methylene blue, and polyethylene glycol. Nerve continuity was assessed post-operative in 5 animals in each group through electron microscopy. Functionality was assessed in the other 10 per group by EMG and whisker analysis after surgery, and weekly for 8 weeks. At 8 weeks, nerves and distal muscles were histologically analyzed. RESULTS/ANTICIPATED RESULTS: PEG fusion technology immediately restored axonal continuity following surgery, demonstrated by electron microscopy. Electrophysiology was also similarly restored across the site immediately, determined through intraoperative nerve stimulation, in the PEG fusion group. The nonintervention group showed dramatically reduced functional recovery than the PEG fusion group following surgery, shown by lower whisking activity and poor electrophysiology outcomes. Furthermore, the PEG fusion group showed statistically significant higher fascicle counts, myelination diameter, axonal diameter, and distal muscle fibers histologically. DISCUSSION/SIGNIFICANCE OF IMPACT: This study demonstrates that polyethylene fusion technology may improve facial reanimation outcomes. PEG is already a FDA-approved drug, and thus the pathway to translational clinical application of this work may thus be streamlined, bringing new options to patients with facial paralysis.

